# SSVEP-based brain–computer interface enabling graded dyspnoea self-report: proof-of-concept study in healthy volunteers

**DOI:** 10.1186/s12984-025-01846-y

**Published:** 2026-01-30

**Authors:** Sébastien Campion, Xavier Navarro-Suné, Isabelle Rivals, Capucine Morélot-Panzini, Laure Serresse, Mario Chavez, Alexandre Demoule, Marie-Cécile Niérat, Mathieu Raux, Thomas Similowski

**Affiliations:** 1 Sorbonne Université, INSERM, UMRS 1158, Paris, France; 2 Équipe de statistiques appliquées, ESPCI, Université PSL, Paris, France; 3 Département R3S, Service de Pneumologie, AP-HP, Groupe Hospitalier Universitaire APHP-Sorbonne Université, Hôpital Pitié-Salpêtrière, Paris, France; 4 AP-HP, Groupe Hospitalier Universitaire APHP-Sorbonne Université, Service des Soins Palliatifs, d’Accompagnement et de Soins de Support, Paris, France; 5 Fédération Hospitalo-Universitaire ‘BREATH’, Paris, France; 6 Institut du Cerveau–Paris Brain Institute, Sorbonne Université, Inserm-CNRS, Paris, France; 7 Département R3S, AP-HP, Service de Médecine Intensive et Réanimation, Groupe Hospitalier Universitaire APHP-Sorbonne Université, Hôpital Pitié-Salpêtrière, Paris, France; 8 Département d’Anesthésie et Réanimatio, AP-HP, Groupe Hospitalier Universitaire APHP-Sorbonne Université, Hôpital Pitié-Salpêtrière, Paris, 75013 France; 9 Département R3S, AP-HP, Groupe Hospitalier Universitaire APHP-Sorbonne Université, Hôpital Pitié-Salpêtrière, 47–83 boulevard de l’Hôpital, 75,651 Paris Cedex 13, Paris, 75013 France

**Keywords:** Dyspnoea, Brain–computer interface, Steady-state visual evoked potentials (SSVEP), Mechanical ventilation, Self-report, Nonverbal communication, Respiratory discomfort, Critical care

## Abstract

**Background:**

Mechanically ventilated patients may experience respiratory suffering, which is difficult to assess when verbal communication is impaired. We evaluated the performance of a steady-state visual evoked potential (SSVEP)-based brain–computer interface (BCI) designed to enable self-reporting of dyspnoea in this context.

**Methods:**

Forty-nine healthy volunteers were studied under five respiratory conditions: normal breathing (NB), inspiratory resistive loading (IRL), inspiratory threshold loading (ITL), CO₂ inhalation (CO₂), and a return to NB as wash-out (NBWO). Respiratory discomfort was evaluated using a visual analogue scale (VAS). Two BCIs models were tested: a detection BCI (D-BCI), designed to discriminate between ‘breathing is OK’ and ‘breathing is difficult’, and a quantification BCI in the form of a LED-based analogue scale (LAS), composed of five light-emitting diodes. Visual stimuli were delivered at different frequency sets: 12–15 Hz, 15–20 Hz, and 20–30 Hz for the D-BCI; low frequencies (13–17–19–23–29 Hz) and high frequencies (41–43–47–53–59 Hz) for the LAS. Performance was assessed using receiver operating characteristic (ROC) curves; the area under the ROC curve (AUC) was the primary outcome.

**Results:**

Participants reported significant respiratory discomfort during IRL, ITL, and CO₂ conditions in the D-BCI groups, and during ITL and CO₂ in the LAS groups, as reflected by higher dyspnoea VAS scores compared to NB. The best-performing frequency sets were 20–30 Hz for the D-BCI (AUC 0.89 [0.89–0.90]) and low frequencies for the LAS (AUC 0.84 [0.83–0.85]).

**Conclusions:**

This study demonstrates that an SSVEP-based BCI can sucessfully detect and quantify experimentally induced dyspnoea in healthy individuals. Further research is needed to evaluate its clinical applicability for assessing dyspnoea in non-communicative patients.

**Supplementary Information:**

The online version contains supplementary material available at 10.1186/s12984-025-01846-y.

## Background

Dyspneoa signals an ‘*upsetting or distressing awareness of breathing sensations*’ [1]. It reflects the brain’s response to abnormal respiratory-related afferents, together with changes in breathing patterns, autonomic reactions, and emotion-related behavioural displays [1, 2]. Dyspnoea attests to intense suffering that demands immediate attention—a primary concern for healthcare professionals that is grounded in moral obligations and fundamental human rights [3]. There is broad consensus that self-report must take precedence to identify, characterise, and treat dyspnoea [2], ensuring that patients are not denied appropriate care when their lived experience diverges from observable or measurable variables [4]. Upholding self-report enacts the principle of epistemic justice—recognising the patient as a credible knower of their own condition and preserving their autonomy within the decision-making process [5].

Verbal self-report is the most natural and straightforward means for patients to engage with caregivers about dyspnoea [6]. Yet there are situations where physical barriers to verbal or gestural exchange cannot be overcome even though consciousness and cognition remain intact—for example, in patients with high cervical spinal cord injuries, myopathies, Guillain-Barré syndrome, myasthenia gravis crises, amyotrophic lateral sclerosis, or locked-in syndrome of any origin, or in mechanically ventilated patients with ICU-acquired weakness, whether during the ICU stay or later in weaning units. Although highly specific, these situations are emblematic as they may involve respiratory suffering at its most intense and terrifying because of complete control deprivation [3, 7, 8] }. In such cases, resorting to observational scales validated to assess respiratory-related suffering in non-communicative individuals is preferable to no assessment at all [9–12]. Yet this approach deprives patients who could theoretically self-report, if not for their physical limitations, of a measure of their autonomy.

It is therefore warranted to explore technological solutions capable of addressing this impasse. Brain–computer interfaces (BCIs) are well-suited to access internal states while bypassing motor and behavioural channels [13], and have been used successuflly for various purposes in several of the clinical situations listed above [14–18]. Among them, steady-state visually evoked potentials (SSVEPs) combine temporal resolution, robustness, and compatibility with low-demand paradigms, making them suitable for operational integration into clinical settings, including but not limited to the intensive care unit [19–22]. SSVEP-based brain–computer interfaces (BCIs) enable communication or control by detecting the brain’s electrical response to visual stimuli flickering at specific frequencies, offering a non-invasive and reliable method for translating user intent into actionable commands. However, their performance depends on factors liable to be altered in dyspnoea-associated circumstances [23–25]. For instance, dyspnoea interferes with attentional processes [26, 27], which could compromise the stability or accuracy of SSVEPs. It may also occur alongside substantial head movements [28], for example, when ‘fighting’ a mechanical ventilator, which could interfere with stimulus fixation or introduce signal artifacts.

We tested the hypothesis that SSVEP-based self-report can detect and quantify dyspnoea during experimentally induced dyspnoea in healthy participants. To this end, we developed an SSVEP-controlled visual ranking scale and identified the stimulus frequencies that elicited the strongest SSVEP responses. We tested the hypothesis that SSVEP-based self-report can detect and quantify dyspnoea during experimentally induced dyspnoea in healthy participants. To this end, we developed an SSVEP-controlled visual ranking scale and identified the stimulus frequencies that elicited the strongest SSVEP responses. The study was conceived and designed as an early proof-of-concept, intended as the starting point of a long-range process needed to consider applications in the clinical field.

## Methods

### Participants

**Fig. 1:**
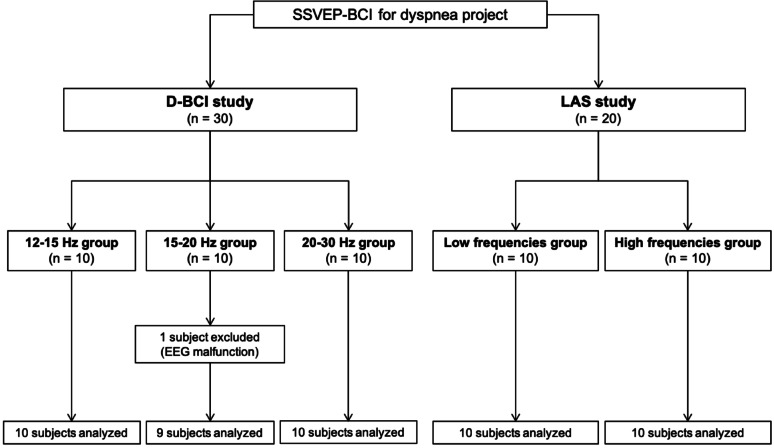
Study flow-chart. *SSVEP-BCI* steady state visual evoked potential-based brain-computer interface, *D-BCI* detection brain-computer interface, *LAS* LED analogue scale, *EEG* electroencephalogram

Fifty healthy volunteers participated in the study (29 women, 21 men, mean age 28 ± 2 years). To be eligible for inclusion, participants had to be at least 18 years old, not placed under legal guardianship, free from any self-reported somatic or psychiatric conditions, and naive to respiratory physiology studies. Non-inclusion criteria mirrored these requirements, with additional disqualifying factors including active smoking or a lifetime tobacco exposure of 5 pack-years or more, as well as pregnancy or breastfeeding. Participants were asked to abstain from analgesics, anti-inflammatory drugs, alcohol, caffeine, and psychotropic substances for 48 h before the experiment, and to avoid sleep deprivation. The study population was then divided into five groups of ten, based on the type of BCI and frequency set used (see below for details). One subject was excluded from the analysis due to EEG malfunction (Fig. 1).

### Ethical considerations

The study was approved by the appropriate ethics and regulatory committee in accordance with French law (Comité de Protection des Personnes Ile-de-France 6, Paris, France). All participants received detailed information about the study and provided written informed consent prior to inclusion. Notably, they were informed of the potential for negative psychological reactions following the induction of experimental dyspnoea and were offered the option of support should such responses occur [29].

### Brain computer interfaces

**Fig. 2:**
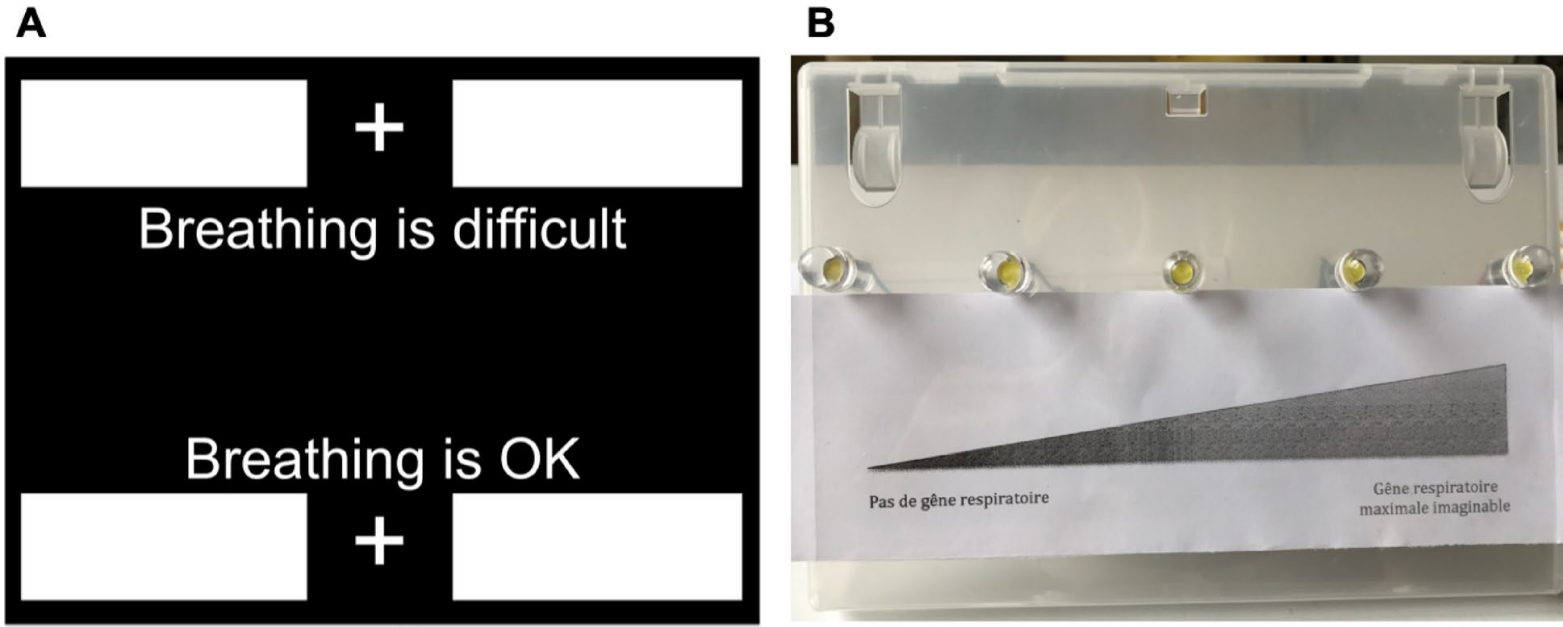
Repetitive visual stimuli for **A** the detection brain-computer interface (D-BCI) and **B** for the LED analogue scale (LAS). ‘*Pas de gêne respiratoire*’ no respiratory discomfort, ‘*Gêne respiratoire maximale imaginable*’ maximum imaginable respiratory discomfort

Two BCIs were tested in this study. The first, a detection BCI (D-BCI), was designed to identify dyspnoea in a dichotomous manner. Two descriptors of breathing sensations (‘*Breathing is OK*’ and ‘*Breathing is difficult*’) were used as repetitive visual stimuli and displayed on a cathode ray tube monitor (Fig. 2a). Three pairs of non-harmonic frequencies were tested: 12–15 Hz, 15–20 Hz, and 20–30 Hz. The second model was developed for the graded assessment of dyspnoea using a light-emitting diode-based analogue scale (LAS). The LAS consisted of five horizontally aligned LEDs, mimicking a numerical rating scale ranging from ‘0’ on the far-left LED (‘no respiratory discomfort’) to ‘4’ on the far-right LED (‘maximum imaginable respiratory discomfort’) (Fig. 2b). Two sets of non-harmonic frequencies were tested: a ‘low-frequency’ set (13, 17, 19, 23, and 29 Hz; LAS_low_) and a ‘high-frequency’ set (41, 43, 47, 53, and 59 Hz; LAS_high_) (see details in electronic supplement ES1). Participants were divided into five groups of ten: D-BCI_12–15_, D-BCI_15–20_, D-BCI_20–30_, LAS_low_, and LAS_high_.

### Data acquisition

#### Respiratory measurements

Participants breathed through a mouthpiece while wearing a nose clip, fitted with an antibacterial filter (Anest-Guard, Teleflex Medical, Athlone, Ireland). A non-compliant tube connected to a linear differential pressure transducer (Validyne^®^ DP15-32, Validyne, Northridge, CA, USA) was used to record airway pressure. Airway flow was measured using a pneumotachograph (Hans Rudolph Inc., USA) connected to a second linear differential pressure transducer (Validyne^®^ DP15-38, Validyne, Northridge, CA, USA). End-tidal carbon dioxide (ETCO₂) was recorded using a capnograph (Nellcor N-85™, Tyco Healthcare Group, Pleasanton, CA, USA). All ventilatory signals were amplified and filtered, then digitised at a sampling rate of 100 Hz (PowerLab, AD Instruments, Hastings, UK).

#### Electroencephalographic recordings

Scalp electroencephalographic (EEG) signals were recorded using an ActiCap^®^ helmet (BrainProducts GmbH, Germany), applied after cleansing the scalp with alcohol. Seven electrodes were positioned according to the international 10–20 system: the reference at FCz, the ground at AFz, three occipital electrodes at O1, O2, and Oz (visual cortex), and one on each earlobe (A1 and A2). A water-based conductive gel was used to ensure electrode impedance remained below 5 kΩ. EEG data were acquired using a 32-channel amplifier (BrainAmp MR^®^, BrainProducts GmbH, Germany), and raw signals were digitised at 2 kHz using BrainVision Recorder^®^ software (BrainProducts GmbH, Germany).

#### Assessment of dyspnoea

Participants rated their level of dyspnoea unidimensionally using a horizontal visual analogue scale (VAS) ranging from 0 to 100 mm, anchored at ‘no respiratory discomfort’ (left) and ‘maximum imaginable respiratory discomfort’ (right). They also completed a post hoc questionnaire describing the qualitative features of their respiratory sensations [30] and previously validated in French [31].

### Protocol

#### Induction of dyspnoea

Mechanically ventilated patients experiencing dyspnoea most often describe their discomfort as excessive inspiratory effort and/or air hunger [8] We therefore employed experimental paradigms known to evoke these specific sensations [see, for example 32]. Dyspnoea of the ‘excessive inspiratory effort’ type was induced using mechanical inspiratory loading techniques: inspiratory resistive loading (IRL) set at 50 cmH₂O·L⁻¹·s⁻¹ (Model #7100, Hans Rudolph Inc., Kansas, USA) and inspiratory threshold loading (ITL) set to 50% of the participant’s maximal inspiratory pressure, calculated as the mean of three standard maneuvers (PowerBreathe© Classic, PowerBreathe Ltd, UK). Dyspnoea of the ‘air hunger’ type was induced by having participants inhale a 5% CO₂ / 95% O₂ gas mixture (CO₂ condition), delivered through a tube connected to the breathing circuit. Analysis of the first group to complete the experiment (D-BCI_15–20_) showed that participants did not experience respiratory discomfort during the CO₂ condition (see details in electronic supplement ES2). As a result, a Douglas bag was added to the circuit in subsequent groups to create a rebreathing effect [as described in 33] The CO₂ condition was excluded from analysis for the D-BCI_15–20_ group.

#### Experimental sequence

Participants were seated in a comfortable chair with armrests, in an electrically shielded room, facing the BCI interface. The experimental sequence consisted of five 5-minute epochs: (1) normal breathing without constraint (NB), (2) inspiratory resistive loading (IRL), (3) inspiratory threshold loading (ITL), (4) hypercapnia via CO₂ inhalation, and (5) a wash-out period with return to normal breathing (NBWO). The experiments always began with epoch 1 and ended with epoch 5, epochs 2, 3, and 4 being presented in random order.

**Fig. 3:**
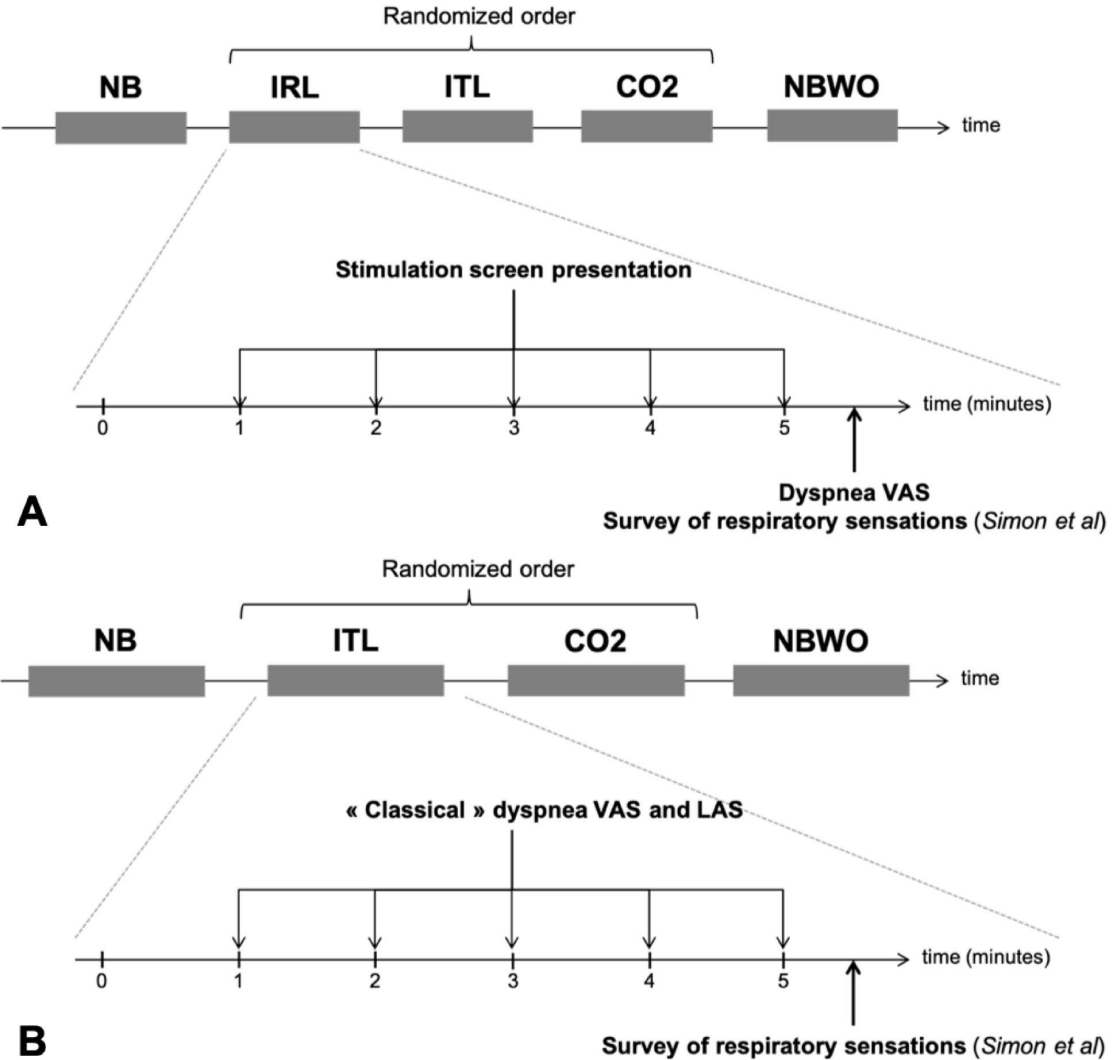
**A** Experimental procedures followed for the validation of the dyspnoea detection brain-computer interface (D-BCI). **B** Experimental procedures followed for the validation of the dyspnoea rating brain-computer interface (LED analogue scale, LAS). *NB* normal breathing without loading, *IRL* inspiratory resistive loading, *ITL* respiratory threshold loading, *CO*_*2*_ carbon dioxide inhalation, *NBWO* NB wash-out, *VAS* visual analogic scale

During each epoch, the D-BCI or LAS system was activated for 15 s every minute (Fig. 3). When the D-BCI was active, participants were instructed to fix their gaze on the fixation cross beneath the sentence that best described their respiratory sensations. After each D-BCI presentation, they reported the sentence they had focused on by pointing to it on the screen. At the end of each condition, the mean level of respiratory discomfort during the epoch was evaluated using a VAS.

When the LAS was active, participants were told to fix their gaze on the LED that best represented the intensity of their respiratory discomfort. After each LAS presentation, they identified the corresponding LED by pointing to it, and rated their discomfort using a horizontal linear potentiometer (MLT1601/ST Response Meter, AD Instruments, Hastings, UK) serving as a VAS. For both the D-BCI and LAS protocols, participants completed Simon’s questionnaire following each condition.

### Data analysis

#### Respiratory data analysis

Respiratory data were analysed and averaged for each condition using LabChart^®^ v7 (AD Instruments, Castle Hill, Australia). The variables of interest were respiratory rate, tidal volume, inspiratory airway pressure, and end-tidal CO₂ (ETCO₂).

#### Electroencephalographic recordings analysis

EEG recordings were preprocessed and analysed offline using MATLAB^®^ (v7.13.0, MathWorks Inc., Natick, MA, USA). Signals from the occipital channels (O1, O2, and Oz) were re-referenced to the earlobe electrodes (A1 and A2) and downsampled to 250 Hz. High-frequency noise and baseline drift were removed using a bandpass filter (1–120 Hz), and power line interference was minimised with a notch filter centred at 50 Hz. EEG data were then segmented into 3-second sliding windows with 50% overlap.

Each preprocessed epoch was spatially filtered to extract a feature—defined as a scalar value representing the strength of the visually evoked potential elicited at a given stimulus frequency (f). Canonical correlation analysis (CCA) was selected for spatial filtering due to its efficiency and robustness [34, 35] CCA is a multivariate statistical method that assesses the correlation between the participant’s EEG signals and reference signals (sinusoidal templates corresponding to the flickering frequencies). CCA coefficients were computed for each frequency, and the highest coefficient was interpreted as the SSVEP response [34].

A feature vector for each frequency was constructed using the correlation coefficients across epochs (t). This was then used to compute a receiver operating characteristic (ROC) curve based on binary labels indicating whether the participant was focusing on the stimulus at frequency f during epoch t (1 = yes; 0 = no).

### Statistical analysis

The primary endpoint was the area under the ROC curve (AUC) for each BCI group. ROC curves were designed and compared according to DeLong’s method [36].

The correlation between VAS and LAS was evaluated using a linear mixed model represented as $\:VAS\sim1+LAS+\left(1\left|subject\right.\right)$, where *LAS* was considered as a fixed effect factor and *subject* as a random effect factor. The model was adjusted using the maximum likelihood. A dyspnoea VAS ≥ 40 mm (out of 100 mm) was considered to reflect significant respiratory discomfort. Similarly, the LAS cut-off value was set to 1.6 (40% of a 0–4 scale), rounded off to 2, as the LAS is an ordinal scale with integers. A LAS ≥ 2 was therefore considered to reflect significant respiratory discomfort and the performance of the LAS to detect significant respiratory discomfort defined as a VAS ≥ 40 mm was tested. Sensitivity (Se), specificity (Spe), positive predictive value (PPV), negative predictive value (NPV) and likelihood ratio (LR) were calculated.

One-way comparisons of more than two modalities were performed using Kruskal-Wallis, Friedman or Skillings-Mack tests, as appropriate, followed by Dunn’s *post-hoc* pairwise multiple comparisons tests when appropriate. Comparisons of only two modalities were performed using Mann-Whitney’s test, exact Fisher’s test or Wilcoxon’s matched-pairs signed rank test, as appropriate. Results were expressed as median and interquartile range [IQR], numbers and percentage or value and 95% confidence interval (95%CI), as appropriate.

Statistical tests were performed using MatLab^®^’s Statistics Toolbox (MathWorks Inc, Natick, MA, USA), Prism^®^ 6.0c (GraphPad Software Inc, La Jolla, CA, USA) and XLSTAT^®^ (Addinsoft, France). The type I error risk was set at 0.05.

## Results

### D-BCI study

#### Response to dyspnoea induction

**Fig. 4:**
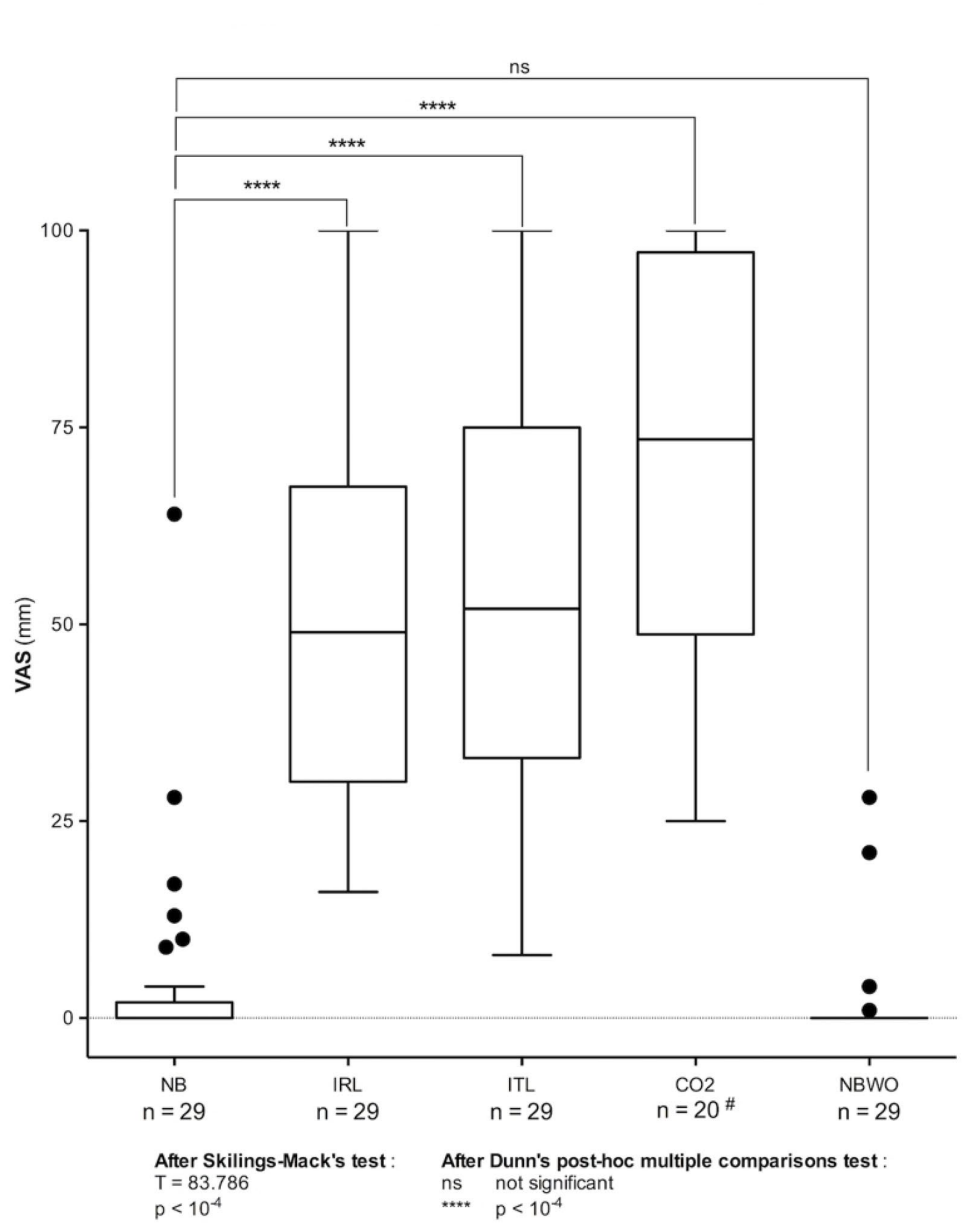
Dyspnoea ratings (visual analogue scale, VAS) during the dysppnea detection brain-computer interface experiments. ^*#*^*after exclusion of data from the D-BCI*_*15-20*_ *group. NB* normal breathing without loading, *IRL* inspiratory resistive loading, *ITL* respiratory threshold loading, *CO*_2_ carbon dioxide inhalation, *NBWB* normal breathing wash-out

Respiratory variables are presented in Table 1. Dyspnoea VAS scores were higher during IRL (49 mm [30–68]), ITL (52 mm [33–75]), and CO₂ (74 mm [49–97]) compared to NB (0 mm [0–2], *p* < 10⁻⁴) (Fig. 4). 79% of participants reported at least one descriptor associated with excessive inspiratory effort during IRL and ITL, while 83% reported at least one descriptor associated with air hunger during CO₂. Both sensations were reported by 38% of participants during IRL, 66% during ITL, and 50% during CO₂. Breathing was perceived as requiring more concentration by 69%, 79%, and 50% of participants during IRL, ITL, and CO₂, respectively.

**Table 1:** Ventilatory variables for the detection brain-computer interface (D-BCI) groups

	NB (*n* = 29)	IRL (*n* = 29)	ITL (*n* = 29)	CO_2_ (*n* = 20) ^#^	NBWO (*n* = 29)
V_T_ (mL)	530 [406–646]	925 ** [677–1314]	954 *** [800–1282]	1655 *** [1473–1900]	491 [391–731]
RR (min^− 1^)	13 [10–16]	7 ** [5–11]	10 [6–12]	15 [12–18]	14 [11–18]
P_aw_ (cmH_2_O)	−1.0 [−1.4–0.8]	−11.4 **** [−18.0–7.9]	−38,9 **** [−64.4—31.15]	−4.0 *** [−5.2–3.3]	−1.1 [−1.5–0.8]
E_T_CO_2_ (mmHg)	38.5 [35.5–40.3]	40.0 [37.8–43.1]	39.8 [33.5–42.8]	53.7 **** [53.0-54.2]	37.0 [33.6–38.8]

*NB* normal breathing without loading, *IRL* inspiratory resistive loading, *ITL* inspiratory threshold loading, *CO2* CO_2_ inhalation, *NBWO* NB wash-out.

^#^after exclusion of data from the D-BCI_15 − 20_ group.

Comparison with NB by Skilings-Mack test and post-hoc Dunn tests: **p < 0.01, ***p < 0.001, ****p < 10^− 4^.

#### EEG analysis and BCI performance

**Fig. 5:**
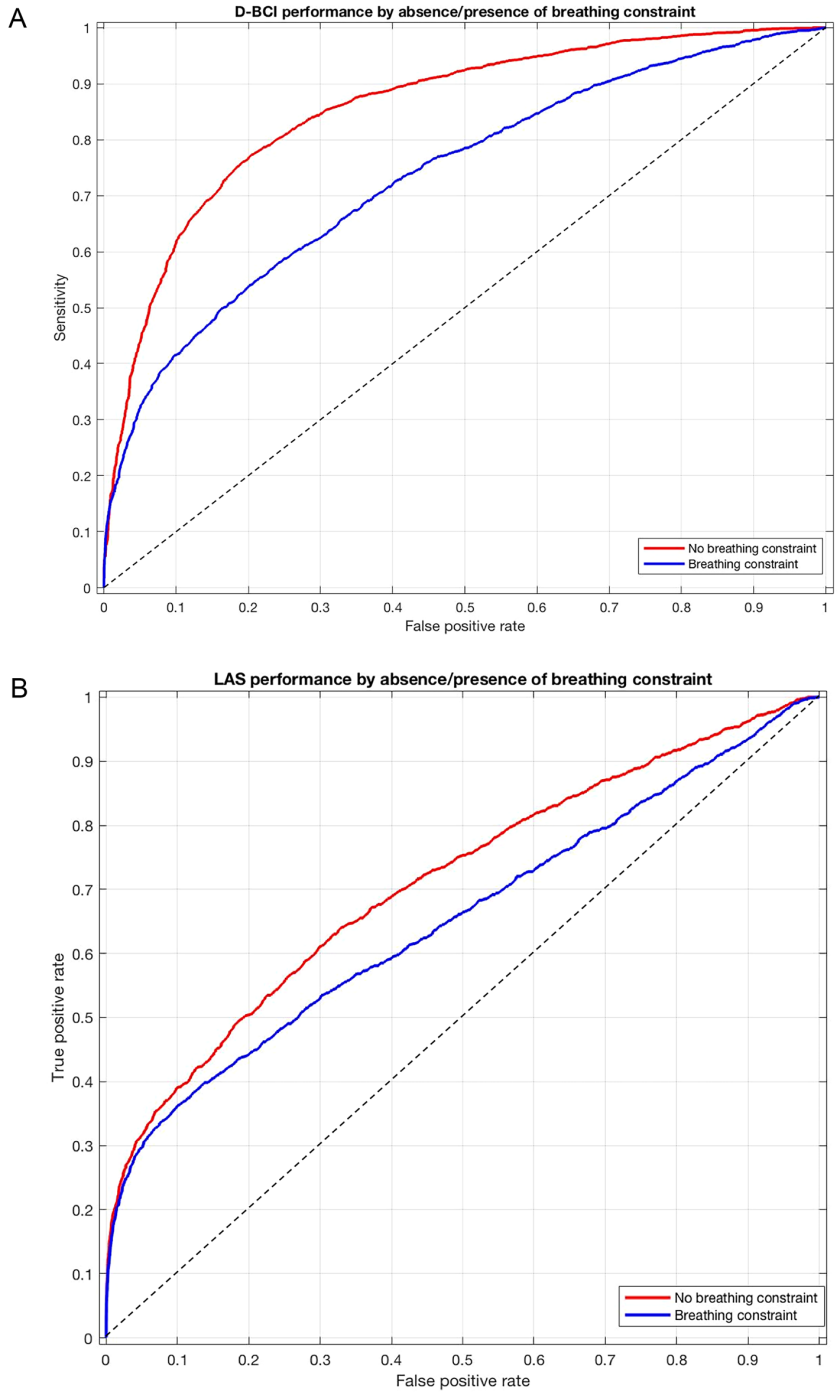
**A** ROC curves describing the performance of the dyspnea detection brain-computer interface (D-BCI). Red curve: in the absence of breathing constraint (normal breathing and normal breathing wash-out condition): AUC 0.86 [0.85-0.86]. Blue curve: in the presence of breathing constraint (resistive and threshold loading, CO_2_ stimulation): AUC 0.74 [0.72-0.75]. **B** ROC curves describing describing the performance of the dyspnoea rating brain-computer interface (LED analogue scale, LAS). Red curve: in the absence of breathing constraint (normal breathing and normal breathing wash-out condition): AUC 0.71 [0.63-0.67]. Blue curve: in the presence of breathing constraint (resistive and threshold loading, CO_2_ stimulation): AUC 0.65 [0.63-0.67]

The D-BCI performed better in the absence of respiratory constraint: the AUC was 0.86 (95% CI [0.85–0.86]) during unconstrained breathing (NB and NBWO) versus 0.74 (95% CI [0.72–0.75]) under respiratory constraint (IRL, ITL, and CO₂) (*p* < 10⁻⁴) (Fig. 5A). Performance under ITL and IRL (AUC 0.79, 95% CI [0.77–0.81]) was lower than under CO₂ (AUC 0.85, 95% CI [0.85–0.88], *p* < 10⁻⁴). The signal-to-noise ratio (defined, for each stimulus frequency, as the ratio between the power at the target flickering frequency and its harmonics when applicable and the average power of neighbouring frequency bins, which served as an estimate of background noise) was also significantly reduced under respiratory constraint (1.23 [1.02–2.5]) compared to the unconstrained condition (2.54 [1.53–4.05], *p* < 10⁻⁴). At baseline, signal-to-noise ratios were higher for D-BCI_12–15_ (3.8 [3.0–5.9]) than for D-BCI_15–20_ (2.0 [1.6–2.9], *p* = 0.051) and D-BCI_20–30_ (1.5 [1.2–2.6], *p* < 0.01).

The D-BCI achieved its best performance with the 20–30 Hz frequency pair: AUC was 0.89 (95% CI [0.89–0.90]) for D-BCI_20–30_, significantly higher than D-BCI_12–15_ (0.86, 95% CI [0.85–0.87], *p* < 0.001) and D-BCI_15–20_ (0.80, 95% CI [0.79–0.81], *p* < 10⁻⁴).

### LAS study

#### Response to dyspnoea induction

**Fig. 6:**
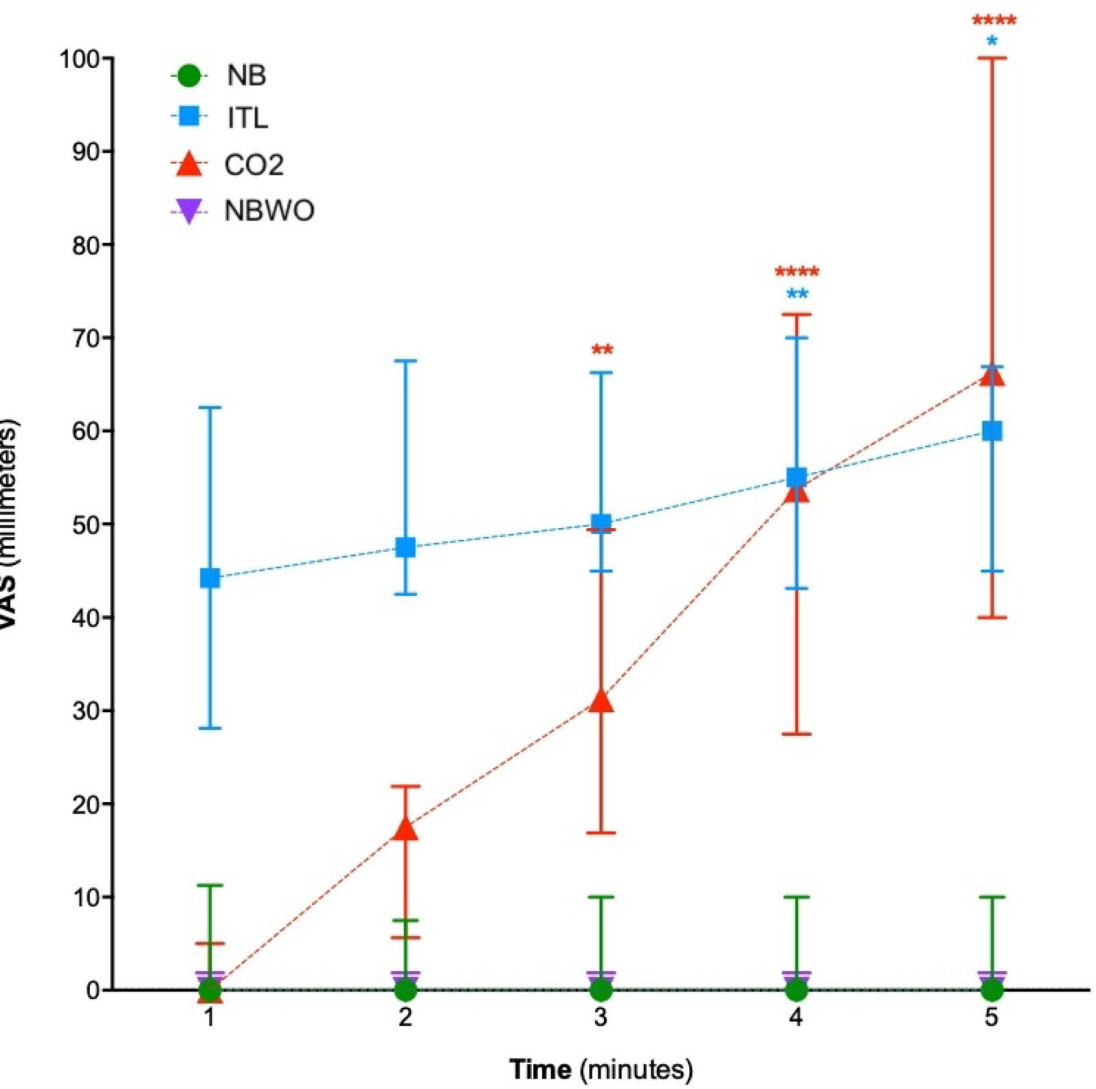
Evolution of dyspnoea ratings (visual analogue scale, VAS) over time in the different experimental conditions during the dyspnoea rating brain-computer interface experiments. NB normal breathing without loading, *ITL* respiratory threshold loading, *CO*_2_carbon dioxide inhalation, *NBWO* spontaneous breathing wash-out. The data points represent median values; the error bars delineate first quartile-third quartile range. *: *p* < 0.05; ** *p* < 0.01; **** *p* < 0.0001 (blue for ITL-SB comparisons, red for CO2-SB comparisons)

Respiratory variables are presented in Table 2. Dyspnoea VAS scores were higher during ITL (53 mm [41–66]) and CO₂ (38 mm [20–45]) compared to NB (2 mm [0–9]; *p* < 10⁻⁴), and increased over time during both ITL and CO₂ conditions (Fig. 6). All participants reported at least one descriptor associated with excessive inspiratory effort during ITL, while 55% reported at least one descriptor associated with air hunger during CO₂. Both sensations were reported by 60% of participants during ITL and by 30% during CO₂. Breathing was perceived as requiring more concentration by 80% of participants during ITL and 20% during CO₂.

**Table 2:** Ventilatory variables for the LED analog scale (LAS) groups

	NB (*n* = 20)	ITL (*n* = 20)	CO_2_(*n* = 20)	NBWO (*n* = 20)
V_T_ (mL)	611 [520–711]	857 ** [653–1175]	1640 **** [1338–2526]	658 [538–806]
RR (min^− 1^)	14 [11–16]	8 **** [6–12]	14 [11–16]	14 [11–16]
P_aw_ (cmH_2_O)	−0.8 [−1.0–0.7]	−37.7 **** [−41.0–36.8]	−2.7 *** [−3.7–2.1]	−0.9 [−1.4–0.7]
E_T_CO_2_ (mmHg)	42 [39–44]	45 [40–47]	55 **** [53–57]	40 [38–41]

*NB* normal breathing without loading, *IRL* inspiratory resistive loading, *ITL* inspiratory threshold loading, CO_2_*CO*_*2*_ inhalation, *NBWO* NB wash-out.

Comparison with NB by Friedman test and post-hoc Dunn tests: ** *p* < 0.01, *** *p* < 0.001, **** *p* < 10^− 4^.

#### LAS-VAS correlation

**Fig. 7:**
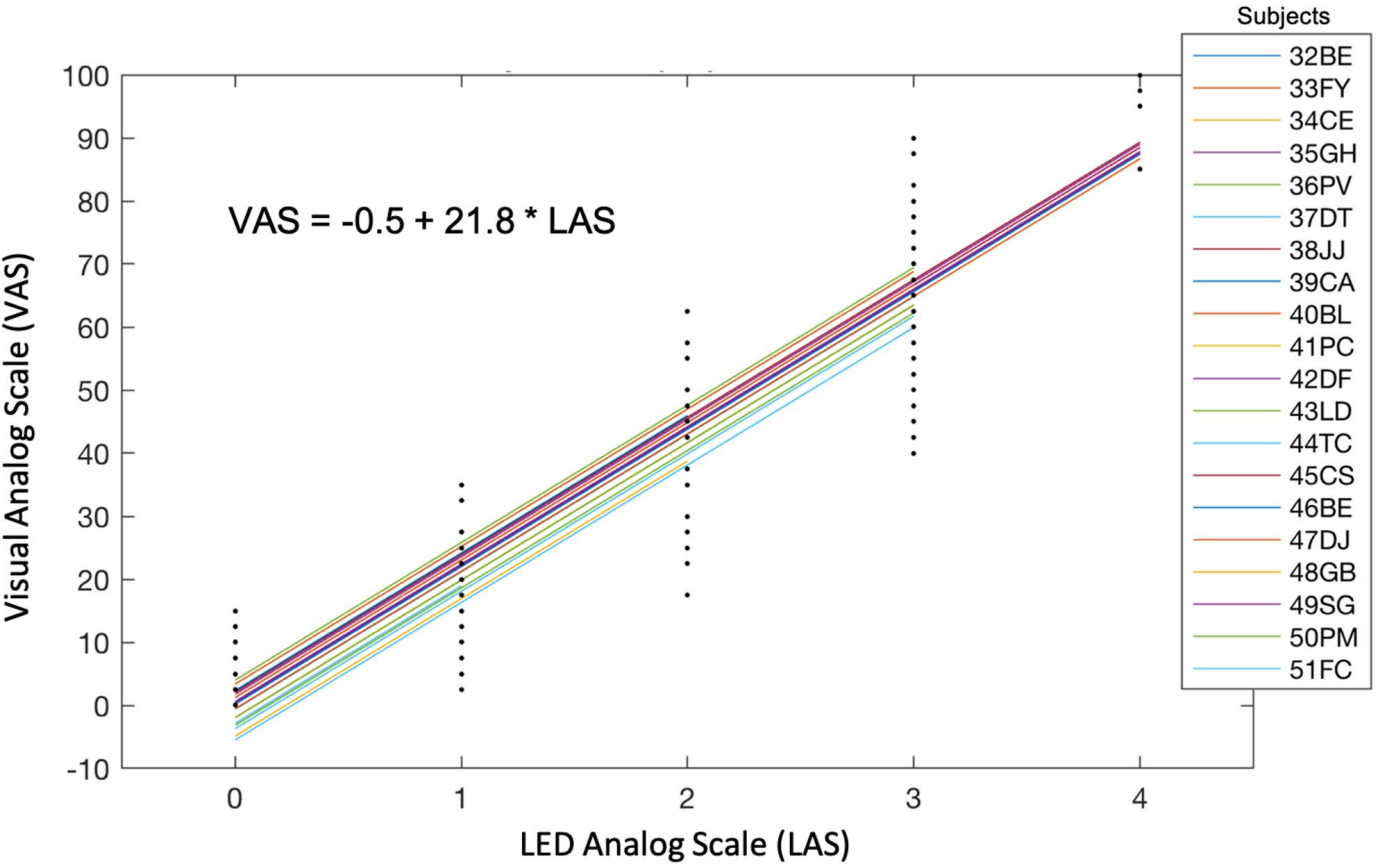
Correlation between visual analogue scale (VAS) and LED analogue scale (LAS) during the dyspnoea rating brain-computer interface experiments, as evaluated using a linear mixed model represented as $\:VAS\sim1+LAS+\left(1\left|subject\right.\right)$, where *LAS* was considered as a fixed effect factor and *subject* as a random effect factor. The model was adjusted using the maximum likelihood

LAS and VAS were highly linearly correlated, as demonstrated by the linear mixed model equation VAS = *a* × LAS + *b*, with *a* = 21.81 (95% CI [21.27–22.36], *p* = 0) and *b* = − 0.48 (*p* = 0.55) (Fig. 7). These coefficients were close to the ideal values of 25 and 0, respectively, which would reflect perfect concordance between VAS and LAS (i.e., VAS = 25 × LAS, with VAS ranging from 0 to 100 and LAS from 0 to 4). The random effect on the intercept was characterised by a standard deviation of 2.94 (95% CI [1.99–4.34]). A LAS ≥ 2 performed well in identifying respiratory discomfort defined by a dyspnoea VAS ≥ 40 mm, with sensitivity 1.00 (95% CI [0.97–1.00]), specificity 0.95 (95% CI [0.92–0.97]), positive predictive value 0.90 (95% CI [0.83–0.94]), negative predictive value 1.00 (95% CI [0.99–1.00]), and likelihood ratio 19.79.

#### EEG analysis and BCI performance

The LAS performed significantly worse under respiratory constraint (ITL and CO₂) than without constraint (NB and NBWO): AUC was 0.65 (95% CI [0.63–0.67]) versus 0.71 (95% CI [0.70–0.73], *p* < 10⁻⁴), respectively (Fig. 5B). LAS performance did not significantly differ between ITL and CO₂ for LAS_low_ (AUC 0.83 and 0.82, respectively, *p* = 0.28), but performance was better during ITL than CO₂ for LAS_high_ (AUC 0.64, 95% CI [0.61–0.67] versus 0.57, 95% CI [0.54–0.60], *p* < 10⁻⁴). Signal-to-noise ratios were significantly lower under respiratory constraint (1.08 [0.45–2.44]) compared to the unconstrained condition (1.97 [0.84–5.00], *p* < 10⁻⁴). During NB, the signal-to-noise ratio was significantly lower for LAS_high_ (0.85 [0.63–0.91]) than for LAS_low_ (4.9 [3.6–9.3], *p* < 10⁻⁴).

The LAS achieved its best performance with the low-frequency set: AUC was 0.84 (95% CI [0.83–0.85]) for LAS_low_ and 0.59 (95% CI [0.57–0.60]) for LAS_high_ (*p* < 10⁻⁴).

## Discussion

We developed and tested a brain–computer interface based on steady-state visual evoked potentials (SSVEPs) to enable nonverbal, nongestural self-report of respiratory discomfort. In healthy participants, the system proved feasible and reliable across experimental conditions known to induce dyspnoea. Beyond enabling participants to signal dyspnoea, this BCI also allowed them to rate the intensity of their respiratory discomfort using a visual ranking scale—an original feature with potential clinical applications. Together, these preliminary findings support the potential of SSVEP-based BCIs as a means of restoring self-report when conventional communication is compromised and justify future efforts to progress toward a clinically usable version.

### Robustness under dyspnoea and attentional stress

To our knowledge, this is the first study to assess the robustness of an SSVEP-based brain–computer interface (BCI) under conditions of acute respiratory discomfort—an essential prerequisite for its potential application in critically ill patients [22]. Experimental dyspnoea induced by inspiratory mechanical loads is known to trigger stress [37], interfere with attentional processes [26], and alter brain activity [26, 38, 39]—all factors that can degrade BCI performance. This was indeed the case in our study, consistent with prior findings on the detrimental effects of divided attention [23, 40, 41] and movement artifacts [35, 42]. In contrast, dyspnoea induced by carbon dioxide inhalation with a hindered ventilatory response does not engage the same cortical networks as inspiratory loading [33], which may explain the relatively preserved BCI performance under hypercapnia. However, this type of experimental dyspnoea is highly aversive and induces intense stress, which may have impaired cognitive performance in our participants [43], possibly in addition to the direct effects of acute hypercapnia [44, 45]. It is important to note that laboratory dyspnoea does not realistically replicate clinical dyspnoea. Healthy participants volunteer to take part, know they are in no real danger, and can stop the experiment at any time, which preserves a sense of control and limits the emotional salience of the experience. The affective dimension of genuine respiratory suffering, whether in the ICU or in other clinical settings, is expected to be much more intense than in the laboratory. We believe that this, if anything, would be more likely to protect performance than to degrade it. Neuromuscular weakness should not be a limiting factor for an SSVEP-based BCI, as the paradigm relies primarily on visual attention rather than volitional motor output. Sedation, by contrast, is likely to affect cortical responsiveness and attention. All of these factors will need to be assessed specifically in order to bridge the gap between the current proof of concept and a clinically usable system.

### Comparison with observer-based assessment tools

Although the performance of the BCI decreased under respiratory constraint, it remained comparable to that of observer-based scores when tested against VAS dyspnoea ratings in communicative patients [11, 12]. These scales, which integrate respiratory, cardio-autonomic, and behavioural indicators, show good correlation with dyspnoea VAS scores [9, 11, 46]. Their diagnostic accuracy in clinical settings, as reflected by the area under the ROC curve, typically ranges from 0.75 to 0.83 for detecting dyspnoea VAS ≥ 40 mm. In this regard, our SSVEP-based BCI demonstrated favourable performance, achieving an AUC of 0.89 for the D-BCI in the 20–30 Hz range and 0.84 for the LAS using low-frequency bands.

### Using the BCI as a numerical rating scale

The LAS component of our BCI did not merely classify the presence or absence of discomfort—it allowed participants to rate the intensity of their respiratory sensations on a discrete, ordinal scale. This makes it, to our knowledge, the first application of an SSVEP-based BCI to support quantitative self-report of a complex, multidimensional symptom like dyspnoea. A VAS score ≥ 40 mm is often used as a threshold for respiratory discomfort significant enough to warrant intervention [1]. By analogy, we defined a LAS score ≥ 2 (on a 0–4 scale) as the corresponding threshold. At this cutoff, LAS performance was robust, with a perfect sensitivity of 1.00 and a specificity of 0.90 for detecting respiratory discomfort. The absence of false negatives is especially important in this context, ensuring that patients experiencing dyspnoea are not left unheard. At the same time, a low false positive rate is essential to avoid alarm fatigue and preserve the trust of caregivers in a potential clinical alert system [47]. Altogether, these findings suggest that LAS has the potential to become a viable real-time communication channel in contexts where standard reporting is impossible.

### Frequency optimisation

SSVEP amplitude is known to vary with stimulus frequency, typically peaking around 15 Hz and declining at higher frequencies [24, 48–51]. Our baseline signal-to-noise ratios were consistent with this trend, yet D-BCI performance was highest in the 20–30 Hz range. This unexpected result may be explained by increased false positives near the alpha band (8–13 Hz) [48], particularly under respiratory constraint, where EEG artifacts are more frequent. For LAS, performance was best with low-frequency stimulation, and declined sharply at high frequencies. This may be due to already low baseline signal-to-noise ratios in the LAS_high_ group, which further deteriorated under constraint. Interestingly, previous studies have reported that higher stimulation frequencies, although generating smaller SSVEP amplitudes, may improve visual comfort and reduce fatigue [52]. This may have contributed to the robust D-BCI performance in the 20–30 Hz range. These results highlight the importance of optimising frequency selection not only for signal strength, but also for resistance to interference and usability.

### Study limitations and perspectives to address them

In our proof-of-concept, feasibility-oriented approach, we chose a synchronous SSVEP-based BCI paradigm because of practical advantages, including a high signal-to-noise ratio and stable frequency-specific activity (valuable when increased breathing efforts generate movement artefacts and create suboptimal EEG acquisition conditions), little or no need for user-specific calibration (in contrast to approaches such as motor imagery or P300 spellers), and minimal cognitive and physical demands on the user (essentially limited to gaze fixation). These advantages are counterbalanced by possible usability and tolerance issues, for example the need for prolonged flickering stimulation. Of note, no formal usability questionnaire was administered to our participants, which could have helped clarify this point. In a clinical setting, it would be preferable for users to report discomfort only on an as-needed basis. Future developments should therefore consider asynchronous paradigms or, possibly more appropriately, hybrid architectures combining SSVEP with an auxiliary control signal on the brain-switch model [53], keeping in mind that with such approaches detection performance is generally lower, false-positive rates are higher, and robust operation typically requires additional confirmation mechanisms [54, 55]. Another feature of our BCI that could be viewed as a limitation is its slow time constant, with 15-second stimulation windows required for a choice to be validated. This was a deliberate decision, taken to maximise SSVEP stability under respiratory constraint and to account for the attentional demands imposed by the breathing loads. In contrast with control-oriented BCIs, where speed is of the essence and where information transfer rate is a major judgement criterion, our aim is to signal a clinically meaningful change rather than to enable rapid command execution. Although improving the ability of dyspnoeic patients to communicate their discomfort is essential, this form of communication is not a matter of actual immediacy. The temporal requirements are therefore fundamentally different from those that apply to BCIs intended for rapid device control. Future work will be necessary to determine whether the SSVEP stimulation window can be usefully shortened. Finally, in addition to clinical difficulties (patient fatigue, sedation, fluctuating attention or motivation), EEG recording in real-life clinical environments can be challenging for both technical and tolerability issues. Gel-based EEG systems, as used here, are not optimal for clinical deployment. However, dry-electrode and rapid-application EEG technologies already exist and are increasingly used in applied environments, including at the bedside and even in the ICU [56–58]. Future iterations of our BCI would rely on such solutions. Likewise, visual stimulation protocols can be adapted to minimise perceptual load. This would be one of the advantages of moving toward an asynchronous or brain-switch architecture.

## Conclusion

The BCI developed in this study may represent the first step toward a means to fight the invisibility of dyspnoea in conscious patients deprived of verbal and gestural communication. If the gaps that separate this preliminary work from a clinically usable device can be bridged,

It may also support caregivers in daily practice by enabling more frequent, targeted interactions around dyspnoea. Future studies conducted in more ecologically valid conditions will be necessary to determine whether satisfactory real-life performance can be achieved.

## Supplementary Information

Below is the link to the electronic supplementary material.

Supplementary material 1.

## Data Availability

Complete de-identified data will be provided to any researcher upon reasonable request to one of the co-last authors.

## Abbreviations

AUC: Area under the ROC curve
BCI: Brain–computer interface
CCA: Canonical correlation analysis
CO₂: Carbon dioxide inhalation
D-BCI: Detection BCI
EEG: Electroencephalogram
ETCO₂: End-tidal carbon dioxide
IRL: Inspiratory resistive loading
ITL: Inspiratory threshold loading
LAS: LED-based analogue scale
LR: Likelihood ratio
NB: Normal breathing
NBWO: Normal breathing as wash-out
NPV: Negative predictive value
PPV: Positive predictive value
ROC: Receiver operating characteristic
Se: Sensitivity
Spe: Specificity
SSVEP: Steady-state visual evoked potential
VAS: Visual analogue scale
95%CI: 95% confidence interval
