# Supplementary material for: SSVEP-based brain–computer interface enabling graded dyspnoea self-report: proof-of-concept study in healthy volunteers
Source: J Neuroeng Rehabil. 2026 Jan 30;23:79. doi: 10.1186/s12984-025-01846-y (PMC12930631; doi:10.1186/s12984-025-01846-y)
Supplement: Supplementary file 1 — Supplementary material 1. [file 12984_2025_1846_MOESM1_ESM.docx]

**Electronic supplementary material**

**SSVEP-based brain–computer interface enabling graded dyspnoea self-report: proof-of-concept study in healthy volunteers**

Sébastien Campion^1^, Xavier Navarro-Suné^1^, Isabelle Rivals^1,2,^ Capucine Morélot-Panzin ^1,3^, Laure Serresse^4,5^, Mario Chavez^6^, Alexandre Demoule^1,7^, Marie-Cécile Niérat^1^, Mathieu Raux^1,8†^, Thomas Similowski^1,9†^*

^1^ Sorbonne Université, INSERM, UMRS 1158 Paris, France

^2^ Université PSL, ESPCI, Équipe de statistiques appliquées, Paris, France

^3^ AP-HP, Groupe Hospitalier Universitaire APHP-Sorbonne Université, Hôpital Pitié-Salpêtrière, Service de Pneumologie, Département R3S, Paris, France

^4^ AP-HP, Groupe Hospitalier Universitaire APHP-Sorbonne Université, Service des Soins Palliatifs, d'Accompagnement et de Soins de Support, Paris, France

^5^ Fédération Hospitalo-Universitaire ‘BREATH’, Paris, France

^6^ Institut du Cerveau–Paris Brain Institute, Sorbonne Université, Inserm-CNRS, Paris, France

^7^ AP-HP, Groupe Hospitalier Universitaire APHP-Sorbonne Université, Hôpital Pitié-Salpêtrière, Service de Médecine Intensive et Réanimation, Département R3S, Paris, France

^8^ AP-HP, Groupe Hospitalier Universitaire APHP-Sorbonne Université, Hôpital Pitié-Salpêtrière, Département d'Anesthésie et Réanimatio, F-75013 Paris, France

^9^ AP-HP, Groupe Hospitalier Universitaire APHP-Sorbonne Université, Hôpital Pitié-Salpêtrière, Département R3S, F-75013 Paris, France

^†^Mathieu Raux and Thomas Similowski contributed equally and are co-last authors

**ES1. Development of the brain-computer interfaces**

The principle of the steady-state visual evoked potential (SSVEP—based brain-computer interface (BCI) is to elicit visual evoked potentials by using repetitive visual stimuli. Spectral analysis of the electroencephalographic (EEG) activity of the visual cortex, recorded by occipital electrodes, identifies the frequency of the repetitive visual stimuli to which the subject directs his or her gaze (Fig. S1).


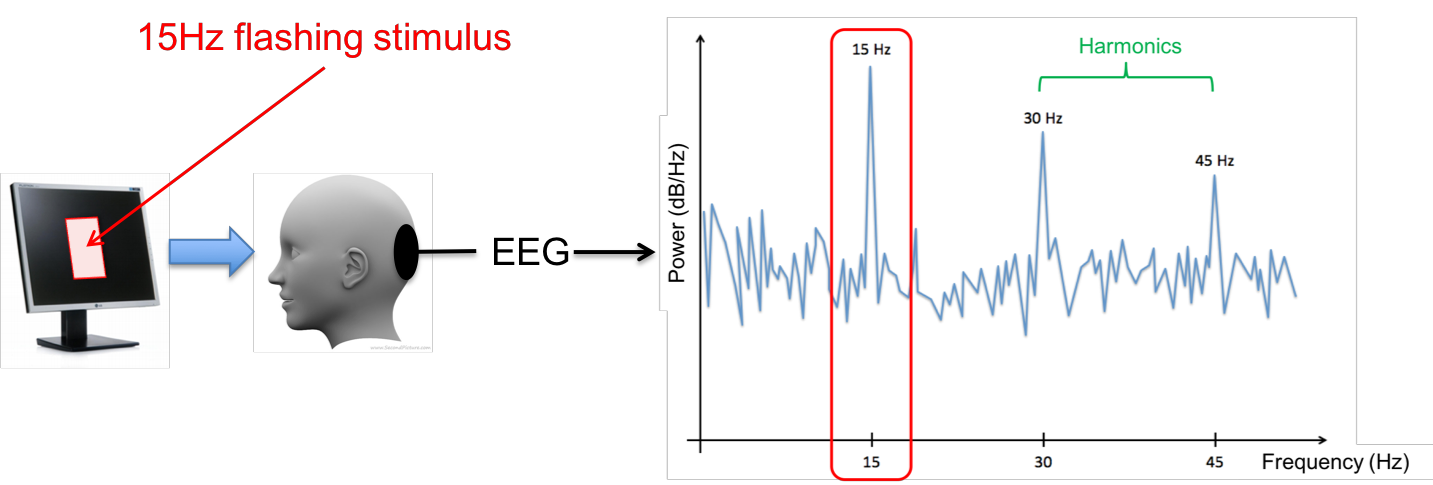


**Fig. S1** – Principle of steady-state visual evoked potentials

Two descriptors of breathing sensations, namely ‘Breathing is OK’ and ‘Breathing is difficult’, were used as repetitive visual stimuli for the detection BCI (D-BCI). They were displayed on a 60 Hz refresh rate cathode ray tube monitor (HPv7650, Hewlett-Packard, Houston, Texas, USA) using Presentation® software (NeuroBehavioral Systems, Berkeley, CA, USA). This software used the refresh rate of the monitor to produce flashing visual stimuli: at each screen refresh, the software either displayed or did not display the stimuli. As the screen refresh rate was 60 Hz, the possible stimuli frequencies were multiples of 60, i.e. 60 Hz (60:1), 30 Hz (60:2), 20 Hz (60:3), 15 Hz (60:4), 12 Hz (60:5), 10 Hz (60:6) etc. To prevent aliasing, the two frequencies had to be non-harmonic to avoid confounding. For example, 15 Hz has a 30 Hz harmonic, so that these two frequencies should not be assigned at the same time. If the BCI identified a 30 Hz SSVEP, it could either be the 2^nd^ harmonic of the 15 Hz stimuli or the fundamental frequency of the 30 Hz stimuli: the BCI is unable to discriminate between these two stimuli and therefore cannot function correctly. We therefore chose three non-harmonic frequency sets: 12-15 Hz, 15-20Hz and 20-30Hz.

For the LED analog scale (LAS), five light-emitting diodes (LED) were aligned horizontally, mimicking a numerical rating scale (NRS). The far-left side LED represented ‘no respiratory discomfort’ with a value of ‘0’. The far-right side represented ‘maximum imaginable respiratory discomfort’ with a value of ‘4’. The values assigned to the other LEDs were ‘1’, ‘2’ and ‘3’ from the left to the right, respectively. The LAS therefore represented a numerical rating scale ranging from 0 to 4. The LEDs were driven by an Arduino^©^ computer card and were separated by a distance of 5° of visual angle, which represents 4.4 cm at a 50 cm viewing distance [48] (Fig. S2). The distance between the subject’s head and the LAS was therefore controlled during the experiments.

As for the D-BCI, frequencies had to be non-harmonic to prevent aliasing. The frequency of each LED was driven by an Arduino^©^ card, which allowed a very precise selection of frequencies. In order to avoid confounding between LEDs, each frequency was chosen as a prime number and the frequency sets were therefore 13 Hz, 17 Hz, 19 Hz, 23 Hz and 29 Hz for the low-frequency set and 41 Hz, 43 Hz, 47 Hz, 53 Hz and 59 Hz for the high frequency set. The lowest frequency was always attributed to the far-left LED and frequencies always increased from left to right (the highest frequency was therefore attributed to the far-right LED).


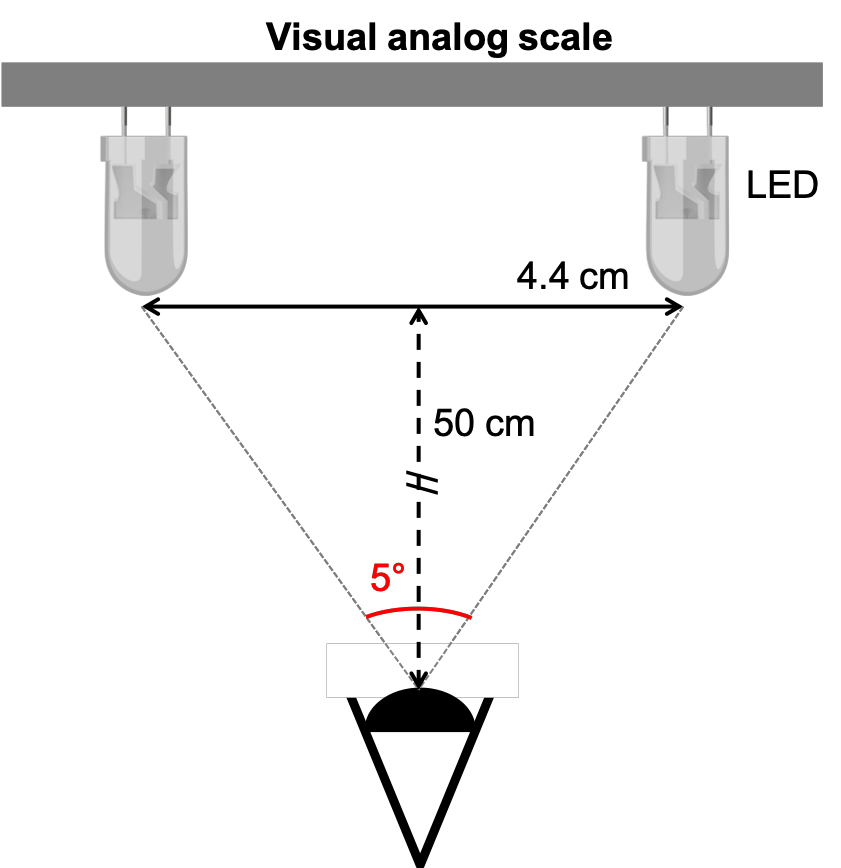


**Fig. S2** – Distance between two LEDs for a 5° visual angle.

**ES2. Carbon dioxide stimulation experiments**

The detection brain-computer interface (D-BCI) 15-20 Hz group (D-BCI_15-20_) was the first to perform the experiment. Under the CO2 condition, these subjects breathed the CO_2_/O_2_ mixture through a tube connected to the breathing circuit. Intermediate analysis of data from this group showed that none of the subjects experienced any respiratory discomfort during the CO2 condition and that median dyspnea visual analog scale (VAS) was not different from that of normal breathing (NB) condition (dyspnea VAS 5 mm [0-22] during CO2 *versus* 0 mm [0-14] during NB, p > 0.99). The CO2 condition was designed to induce hypercapnia, which should have led to increased ventilation to increase the CO_2_ clearance. Dyspnea was expected to occur when the subject’s ventilation reached a maximum, while continuing hypercapnia required increased ventilation [49]. However, only a slight increase in end-tidal CO_2_ partial pressure (E_T_CO_2_) was observed during this condition compared to NB (36 mmHg [31-40] *versus* 39 mmHg [38-44]; p < 0.01). Consequently, tidal volume (V_T_) did not significantly increase during CO2 condition as compared to NB in the D-BCI_15-20_ group (+6% [-29.5-23.5]) and dyspnea was not induced in the absence of specific chemostimulation. This experimental limitation was corrected by modifying the breathing circuit for the subjects of the D-BCI_12-15_ and D-BCI_20-30_ groups: the CO_2_/O_2_ mixture was administered in a Douglas bag through which the subjects inspired and expired. This rebreathing phenomenon induced a significant increase in E_T_CO_2_ between NB and CO2: respectively from 36 mmHg [34-40] to 54 mmHg [53-56] (p < 0.01) for the D-BCI_12-15_ group and from 39 mmHg [37-41] to 54 mmHg [52-55] (p < 0.01) for the D-BCI_20-30_ group, respectively. V_T_ significantly increased in response to hypercapnia in both groups: +211% [175-257] (p < 0.01) for the D-BCI_12-15_ group and +219% [167-270] (p < 10^-4^) for the D-BCI_20-30_ group. Consequently, subjects of the D-BCI_12-15_ and the D-BCI_20-30_ groups experienced respiratory discomfort during the CO2 condition with a significantly higher dyspnea VAS than during NB: 72 mm [64-92] *versus* 0 mm [0-1] (p < 10^-4^) for the D-BCI_12-15_ group and 77 mm [38-100] *versus* 0 mm [0-0] (p < 0.001) for the D-BCI_20-30_ group. The CO2 condition for the D-BCI_15-20_ group was therefore removed from subsequent analyses in the D-BCI study.
